# Protein kinase Ds promote tumor angiogenesis through mast cell recruitment and expression of angiogenic factors in prostate cancer microenvironment

**DOI:** 10.1186/s13046-019-1118-y

**Published:** 2019-03-06

**Authors:** Wanfu Xu, Jiabi Qian, Fangyin Zeng, Songyu Li, Wenjing Guo, Liping Chen, Guihuan Li, Zhishuai Zhang, Qiming Jane Wang, Fan Deng

**Affiliations:** 1 Department of Cell Biology, School of Basic Medical Sciences, Southern Medical University, Guangzhou, 510515 China; 2 Department of Clinical Laboratory, The Fifth Affiliated Hospital, Southern Medical University, Guangzhou, 510900 China; 3 Department of Pharmacology and Chemical Biology, University of Pittsburgh School of Medicine, Pittsburgh, PA 15261 USA; 4 Present address: Guangzhou Institute of Pediatrics, Guangzhou Women and Children’s Medical Center, Guangzhou Medical University, Guangzhou, 510623 China

**Keywords:** Protein kinase D(PKD), Mast cells(MCs), Angiogenesis, Prostate cancer

## Abstract

**Background:**

Mast cells are being increasingly recognized as critical components in the tumor microenvironment. Protein Kinase D (PKD) is essential for the progression of prostate cancer, but its role in prostate cancer microenvironment remains poorly understood.

**Methods:**

The expression of PKD, mast cells and microvessel density were examined by IHC. The clinical significance was determined by statistical analyses. The biological function of PKD and the underlying mechanisms were investigated using in vitro and in vivo models.

**Results:**

PKD2/3 contributed to MCs recruitment and tumor angiogenesis in the prostate cancer microenvironment. Clinical data showed that increased activation of PKD at Ser744/748 in prostate cancer was correlated with mast cell infiltration and microvascular density. PKD2/3 silencing of prostate cancer cells markedly decreased MCs migration and tube formation of HUVEC cells. Moreover, PKD2/3 depletion not only reduced SCF, CCL5 and CCL11 expression in prostate cancer cells but also inhibited angiogenic factors in MCs. Conversely, exogenous SCF, CCL5 and CCL11 reversed the effect on MCs migration inhibited by PKD2/3 silencing. Mechanistically, PKD2/3 interacted with Erk1/2 and activated Erk1/2 or NF-κB signaling pathway, leading to AP-1 or NF-κB binding to the promoter of *scf, ccl5* and *ccl11*. Finally, PKD-specific inhibitor significantly reduced tumor volume and tumor growth in mice bearing RM-1 prostate cancer cells, which was attributed to attenuation of mast cell recruitment and tumor angiogenesis.

**Conclusions:**

These results demonstrate a novel PKDs function that contributes to tumor angiogenesis and progression through mast cells recruitment in prostate cancer microenvironment.

**Electronic supplementary material:**

The online version of this article (10.1186/s13046-019-1118-y) contains supplementary material, which is available to authorized users.

## Background

Prostate cancer (PCa) is the most common diagnosed cancer and accounts for the second most frequent cause of death from cancer in men in western countries [1, 2]. In China, prostate cancer morbidity and mortality are also increasing in the last decade [3]. A growing body of evidence indicated that chronic inflammation mediated by several immune cells, such as macrophages, T cells, neutrophils, and mast cells (MCs) has been shown to play an important role in prostate cancer development and progression [4, 5]. However, the contribution of specific immune cells to the pathogenesis and progression of prostate cancer remained obscure.

Mast cells, which function as immune regulatory cells in allergic reactions and autoimmunity [6, 7], are increasingly recognized as critical components of the tumor stromal microenvironment in a number of human cancers [8, 9]. In many cancers, increased mast cell accumulation has been correlated with poor prognosis [10, 11], angiogenesis, tissue remodeling and immunomodulation. Current data also showed that MCs may exert pro- or anti-tumoral roles, depending on tumor type, microenvironment signals and neighboring interacting cells [9, 12]. In prostate cancer, several studies have tried to address the actual role of MCs in promoting or suppressing cancer, but they did not assess the correlation of mast accumulation with prognosis [13, 14]. Recently, peritumoral MCs were shown to enhance angiogenesis and tumor growth in the orthotopic AT-1 rat prostate tumor model, and MCs were seen recruited in the peritumoral compartment in men during the formation of androgen independent prostate cancer. In contrast, high MCs numbers in the intratumoral compartment were shown to be associated with a favorable outcome in patients [15, 16]. These studies suggest that the actual role of MCs in prostate cancer is indeed context, and regulation of mast cells recruitment and consequent function are needed to be fully deciphered in the prostate cancer microenvironment.

The protein kinase D (PKD) family of serine/threonine kinases, which can be activated by gastrointestinal hormones, comprises three distinct isoforms that modulate a variety of cellular processes. Aberrant PKD activity and expression have been demonstrated in tumor cell lines and pancreatic tumor tissues [17], as well as those from the skin [18], breast [19], and prostate [20]. In particular, PKD has been implicated in many aspects of tumorigenesis and progression, including survival, growth and invasion [20]. Our and other’s lab have shown that PKD plays an important role in the pathogenesis of prostate cancer and targeted PKD inhibition potently blocks cell proliferation and survival in prostate cancer [20, 21]. Recent evidence has shown that PKD isoenzymes regulate vascular endothelial growth factor-A (VEGF-A)-induced endothelial cell proliferation, migration, and angiogenesis [22]. However, whether PKDs regulate tumor angiogenesis through stromal cells in the prostate cancer microenvironment remains unknown.

In this study, we evaluated the role of PKDs in the recruitment of MCs and MCs-mediated angiogenesis in prostate cancer microenvironment. Our study reveals a novel mechanism of PKDs in promoting tumor angiogenesis by enhancing recruitment of MCs and expression of angiogenic factors in the prostate cancer microenvironment.

## Methods

### Cell lines and cultures

The prostate cancer cell lines, PC-3, DU145 and RM1, and the mouse mast cell lines P815 were obtained from the American Type Culture Collection and cultured in Dulbecco’s modified Eagle’s medium (DMEM, Gibco) supplemented with 10% fetal bovine serum (FBS, Gibco) in a humidified incubator at 37 °C with 5% CO_2_. All the cell lines identity was verified before experiments.

### Plasmid and siRNA transfection

The plasmid GFP-C2*,* GFP-PKD1*,* GFP-PKD2 and GFP-PKD3, kindly gifted by Prof. Q. Jane Wang, were transfected into cells transiently by Hilymax (Dojindo, kumamoto, Japan) as suggested by the user manual. siRNA, from GenePharma, was transfected into cells using Lipofectamine 3000 reagent (Invitrogen), according to the manufacturer’s instructions. The siRNA sequence is listed in Additional file 1: Table S1.

### Isolation and culture of bone marrow derived mast cells

C57BL/6 mice were killed and their femurs were obtained in aseptic conditions. Marrow was expelled with culture medium, and bone marrow cells were then washed, spun and cultured in RPMI 1640 supplemented with 10% FBS. The cells were cultured in the presence of IL-3 and SCF (10 ng/mL each, PeproTech, Rocky Hill, NJ) (these cells are referred to here as BMMCs) as described previously [23] .

### Chemotaxis assay

The chemotaxis of P815 MCs was monitored using 24-well with a pore size of 8 μm in chambers. Briefly, the supernatant was added to chambers below of the filter, while P815 MCs was added to upper chambers. After 8 h at 37 °C and in 5% CO_2_, the filters were fixed and stained in a dye solution containing 20% (*v*/v) methanol and 0.1% (*w*/*v*) crystal violet. The cells that had migrated were imaged and counted.

### RNA extraction and quantitative real-time PCR

Total RNA was extracted from cultured cells with TRIZOL reagent (Invitrogen), according to the manufacturer’s protocol. All cDNA samples were prepared using an All-in-one First-stand cDNA synthesis kit (GeneCopoeia, MD, USA). Quantitative real-time PCR (RT-qPCR) analyses were performed with an All-in-one qPCR mix (GeneCopoeia), according to manufacturer’s instructions using an ABI StepOnePlus™ qPCR system. Primers and gene ID are listed in Additional file 1: Table S2.

### Endothelial cell tube formation assay

96-well plates were coated with Matrigel Basement Membrane Matrix (BD Biosciences) and then allowed to polymerize at 37 °C for at least 30 min. HUVECs were treated with conditional medium for 8 h, tubes formation of HUVECs can be visualized and the number of nodes (defined as when at least three cells formed a single point) per image was quantified as described [24].

### Enzyme-linked immunosorbent assays (ELISA)

Quantitative measurement of cytokines, including stem cell factor (SCF), Chemokine ligand 5 (CCL5), C-C motif chemokine 11(CCL11) and vascular endothelial growth factor (VEGF) secreted into conditioned medium was determined using ELISAs, according to the manufacturer’s protocol (BOSTER).

### Immunoprecipitation and immunoblotting assays

Cell lysates were extracted in IP-lysis buffer (50 mM Tris–HCl, pH: 7.5; 150 mM NaCl; 1 mM ethylenediaminetetraacetic acid-2Na (EDTA-2Na); 10% Triton X-100; 0.5 mM Na4P_2_O_7_·10H_2_O; 1 mM C_3_H_7_Na_2_O_6_P_5_(H_2_O); 1 mM Na_3_VO_4_), supernatants were clarified by centrifugation at 12000 g and incubated at 4 °C with the corresponding antibodies overnight, PKD2(CST, 8188,1:2000 for WB), PKD3(CST, 5655,1:2000 for WB), α-tubulin(Ray, RM2007,1:5000 for WB), VEGF(Abclonal, A12303,1:2000 for WB), Erk1/2(Abclonal, A10613,1:2000 for WB), p-Erk1/2(CST, 4370, 1:2000 for WB), p-c-Jun(Ser63)(Bioworld, BS4045; Abclonal, AP0048, 1:2000 for WB), p-c-Jun(Ser73)(Bioworld, BS4046; Abclonal, AP0119, 1:2000 for WB), p-c-Fos(Ser32)(CST, 5348; Immunoway, YP0442, 1:1000 for WB), p65(CST, 8242, 1:2000 for WB), p-p65(Ser536)(CST, 3033, 1:2000 for WB), p-p65(Ser276)(CST, 3037, 1:2000 for WB), p38(CST, 8690, 1:2000 for WB), c-Jun(CST, 9165, 1:2000 for WB), c-Fos(CST, 2250, 1:2000 for WB), after a further 2 h incubation with 20 μl of protein A/G (GE Healthcare). The mix was lysed with 2x laemmli sample buffer and analyzed by immunoblotting to detect proteins. Briefly, the protein was transferred to a 0.22 μm nitrocellulose transfer membrane. The membrane was blocked with 5% (*w*/*v*) milk in PBS/0.05% (*v*/v) Tween-20 and then incubated with the indicated antibody overnight. This was followed by incubation with a horseradish peroxidase secondary antibody (Jackson ImmunoResearch) for 1 h at room temperature. Proteins were detected using enhanced chemiluminescence substrates (Perkin Elmer).

### Chromatin immunoprecipitation (ChIP) assays

ChIP assays were performed in DU145 cells by using an EZ-Zyme Chromatin Prep Kit (Millipore) as pervious work [25]. Briefly, 1.0 × 10^7^ cells were cross-linked with 1% (w/v) formaldehyde for 10 min and then quenched in 0.125 M glycine for 5 min. Cells were lysed and digested to collect the chromatin. IP was carried out by using the indicated antibody overnight. The precipitated DNAs were analyzed and quantified by using real-time PCR analysis. Primer sequences are listed in Additional file 1: Table S3.

### Immunohistochemistry (IHC) of human tissue microarrays

A human prostate cancer tissue microarray was purchased from Alenabio (Additional file 1: Table S4 and Additional file 2: Supplementary data of clinical array). Prostate cancer samples were immunostained against indicated antibodies. Briefly, the slides were dewaxed and rehydrated in distilled water, sections were immersed in citrate buffer(C_6_H_5_Na_3_O_7_·2H_2_O) and then microwaved for 20 min for antigen retrieval. Endogenous peroxidase activity was blocked with 0.5% (*v*/v) H_2_O_2_. The slides were then transferred into a humidified chamber, incubated with 5% (v/v) horse serum for 30 min and then incubated with primary antibodies overnight at 4 °C. After primary antibody CD31(Sangon Biothch, D260721,1:200 for IHC), c-Kit(Sangon Biothch, D155222,1:200 for IHC, phosphor-PKD Ser744/748(CST, 2051, 1:200 for IHC) incubation, the slides were immersed in peroxidase-labeled secondary antibody for 30 min at room temperature. To detect the antibody-conjugated antigen reaction, the sections were incubated in 3-amino-9-ethylcarbazole substrate-chromogen for 30 min and counterstained with hematoxylin.

### In vivo mice experiments

All animal experiments were conducted according to Southern Medical University’s animal institutional regulations and the relevant authorities approved the research protocols. Briefly, 1 × 10^4^ RM1 prostate cancer cells were injected into both flanks of C57BL/6 mice. Once tumors were palpable, mice were randomized into the following groups (5 mice per group): (A) control (vehicle; 5% dextrose); (B) 0.36 mg/kg CRT0066101 and (C) 0.72 mg/kg CRT0066101 (dissolved in 5% dextrose) administered by intraperitoneal injection once daily. Tumor volumes were monitored for the next 2 weeks. After tumors were excised, tumor weight was measured and tumor volume was calculated according to the formula (W^2^ × L)/2, where W is width and L is length of the tumor [26].

### Statistical analysis

All statistical analyses were completed using IBM SPSS Statistics 21. All statistical graphing was completed using GraphPad Prism 6 software. The t test was used to determine the significance of differences in the qPCR assay. *One-way ANOVA* was performed on data from chemotaxis, ELISA assays and endothelial cell tube formation assay. For correlation analysis, the Pearson and *Spearmen test* was used. *p* value of less than 0.05 was considered statistically significant.

## Results

### PKD activation is correlated with microvascular density and MCs recruitment in prostate cancer

**Fig. 1:**
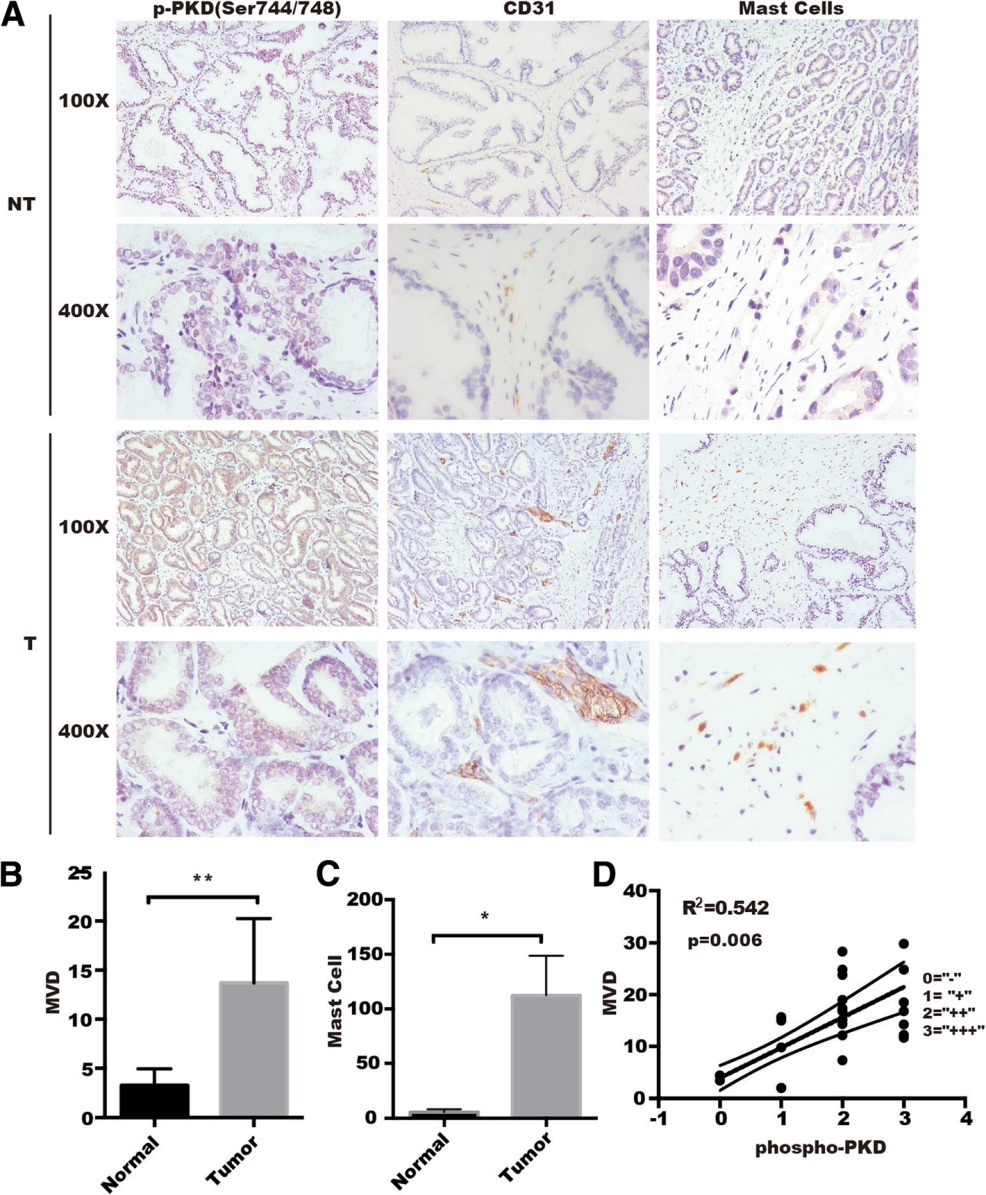
Activation of PKD at Ser744/748 is correlated positively with mast cells infiltration and microvessel density in prostate cancer. **a** Representative images of immunohistochemistry staining for activation of PKD (phospho-PKD Ser744/748), abundance of angiogenesis (CD31 marker) and of mast cells (c-Kit) in prostate cancer tissue and paired control. Quantification of MVD (**b**) and mast cells (**c**) in clinical tissue microarrays between cancer tissues (*n* = 20) and paired controls (*n* = 4) were analyzed by *t-tests*. **p < 0.01, *p < 0.05. **d** A positive correlation was presented between phospho-PKD and MVD

Accumulating evidence demonstrated that tumor-infiltrating activated MCs were significantly associated with progression of solid tumors through various mechanisms including promoting tissue remodeling, immune suppression and angiogenesis [27–29]. We have previously found that PKD1 and PKD3 are upregulated in prostate cancers [20], but another data also showed that PKD1 was downregulated in metastatic prostate cancer [30]. Meanwhile, according to TCGA data [Prostate Adenocarcinoma (TCGA, PanCancer Atlas)], PKD1/2/3 expression in prostate cancer, at mRNA levels, are upregulated in about 4–5% tumors (Additional file 3: Figure S1), suggesting that it is not so much about overexpression or amplification in tumors, the aberrant activation of PKD1/2/3 may plays a more important role in tumor progression. To explore the relationship of PKD activation with MCs recruitment and tumor angiogenesis, we detected the phosphorylation of PKD, microvessel density (MVD), and MCs by IHC in two sets of 24 tissue microarrays of human prostate cancers (Additional file 1: Table S5). As shown in Fig. 1a-c, the phosphorylation of activation loop at s744/748 for PKD (p-PKDser744/748), CD31 (an endothelial cell marker) and c-Kit (a MCs marker) were significantly increased in prostate cancers compared with normal prostate tissue. We then detected autophosphorylation of PKD at s916 by IHC, as shown in Additional file 4: Figure S2, there is no difference for autophosphorylation of PKD at s916 between normal prostate tissue and prostate cancers. Interestingly, the phosphorylation of activation loop at s744/748 for PKD was positively correlated with MVD in prostate cancers compared with normal prostate tissues (Fig. 1d, Additional file 1: Table S5). These data suggested that PKD promoted tumor angiogenesis through regulating MCs and MVD in the tumor microenvironment.

### Inhibition of PKD expression and activity in prostate cancer cells decrease chemotactic migration of MCs

**Fig. 2:**
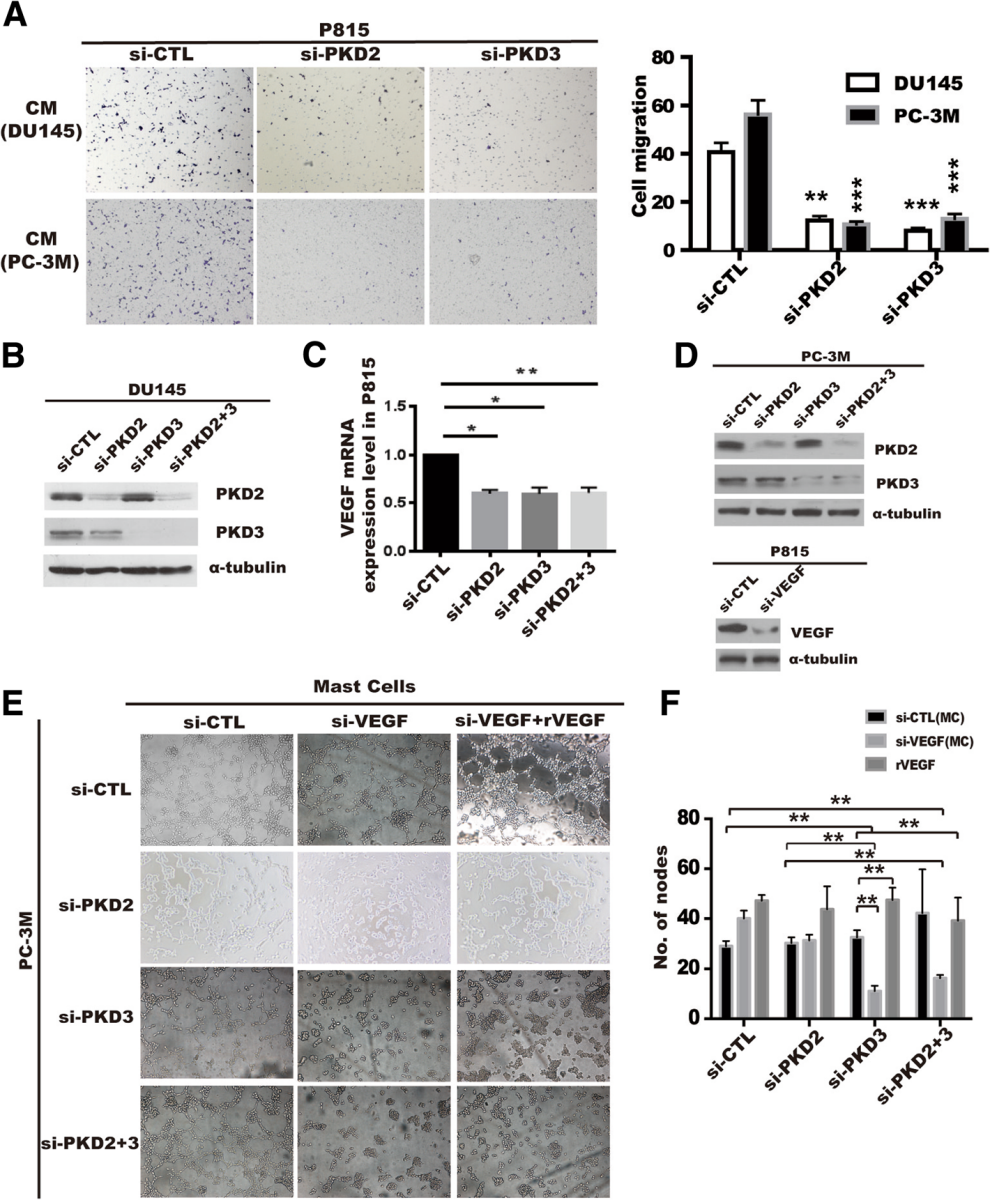
Prostate cancer cells-derived PKD2/3 promote chemotactic migration of mast cells and endothelial cells tube formation. **a** Conditional medium (CM) from PC-3 M or DU145 cells transiently transfected with si-CTL, si-PKD2, si-PKD3 was used to be chemoattractant for P815 mast cell migration by transwell assays. ***p < 0.001, **p < 0.01 versus si-CTL by *one-way ANOVA*. **b** Silencing of PKD2 and PKD3 verification by Western blotting (left panel). **c** VEGF mRNA level was assessed by qPCR in P815 mast cells induced by conditional medium from DU145 cells transfected with siRNA of PKD2 or /and PKD3 (right panel). *p < 0.05, **p < 0.01 versus si-CTL by *one-way ANOVA*. **d** Knockdown efficiency were verified by Western blot in PC-3 M(up) or P815 mast cells(bottom). Cells were transiently transfected with siRNA of indicated genes using lipofectmine3000. **e** HUVEC cells were treated with conditional medium from co-cultured of P815 mast cells transfected with si-CTL or si-VEGF and PC-3 M cells transfected with si-CTL, si-PKD2, si-PKD3 or si-PKD2 + 3. Tube formation of HUVEC cells were visualized by phase contrast inverted microscope (100×). **f** Tube formation was assessed the number of nodes per image was quantified and analyzed by two way ANOVA, **p < 0.01

To explore possible role of PKDs in the MCs recruitment in the prostate microenvironment, we investigated the effect of PKDs in prostate cancer cells on MCs migration using co-cultured system. As shown in Fig. 2a, the conditional medium (CM) from PKD2- or PKD3-depleted PC-3 M or DU145 cells significantly reduced the chemotactic migration of P815 MCs compared with CM from the si-CTL control cells. Similarly, chemotactic migration of P815 MCs (Additional file 5: Figure S3A) or BMMCs (Additional file 5: Figure S3B) isolated from mice were also significantly inhibited by CM from DU145 cells or PC-3 M cells treated with a PKD specific inhibitor kb-NB142–70 [31]. These results suggest that PKD of tumor cells may contribute to the MCs recruitment through specific chemokines secretion in prostate cancer cells.

### PKD2 and PKD3 of prostate cancer cells promote HUVEC cells tube formation via induction of angiogenic factors in MCs

MCs appear at the edges of invasive tumors (peritumoral mast cells) [32, 33] in various tumor models and facilitate angiogenesis at least in part by triggering the release of angiogenic factor VEGF [34]. We have previously demonstrated that PKD2 or PKD3 did not promote angiogenic factor VEGF expression in prostate cancer cells [35], which is confirmed by the ELISA results that depletion of PKD2 or PKD3 has no effect on VEGF secretion in either prostate cancer cells (Additional file 6: Figure S4A-B) or mast cells (Additional file 6: Figure S4C-D). Similarly, the CM from DU145 cells transfected with GFP-PKD2 or GFP-PKD3 failed to alter tube formation in HUVEC cells (Additional file 4: Figure S4E-F). In order to investigate whether PKD2 and PKD3 of prostate cancer cells regulate HUVEC cells tube formation in MCs-dependent manner, we first detected the effect of PKD2 and PKD3 of prostate cancer cells on the angiogenic factors expression in the P815 MCs. PKD2 and PKD3 silencing were verified by Western blotting (Fig. 2b). Compared with control group, mRNA level of VEGF (Fig. 2c), and other angiogenic factors such as TNF-α, IL-6, IL-8 and FGF-2 (Additional file 7: Figure S5) was significantly reduced in the P815 MCs treated with CMs from DU145 cells transfected with siRNA of PKD2 and/or PKD3.

We then explored whether PKD2/3 of prostate cancer cells mediated HUVECs tube formation through upregulation of VEGF expression in mast cells. After transfection with siRNA of PKD2, PKD3 in PC-3 M cells (Fig.  2d, up), and with siRNA of VEGF in P815 MCs (Fig. 2d, bottom), we collected the corresponding supernatant and co-cultured with HUVECs. As expected, the tube formation of HUVECs was not affected by the CM from PC-3 M cells transfected with siRNA of PKD2 and/or PKD3 using co-cultured with MCs (Fig. 2e, left column). However, the tube formation of HUVECs was completely diminished in co-cultured medium of PC-3 M cells transfected with siRNA of PKD2 or PKD3 and mast cells with VEGF silencing (Fig. 2c, middle column of Fig. 2e), which were reversed by the addition of rVEGF into co-culture medium of HUVECs and MCs (Fig. 2E, right column), the corresponding statistics was shown in Fig. 2f. These data indicated that PKDs of prostate cancer cells contributed to the tumor angiogenesis through regulating angiogenic factors secretion of mast cells in prostate cancer microenvironment.

### PKD2 and PKD3 enhance chemotactic migration of mast cells via SCF, CCL5 and CCL11 secretion in prostate cancer cells

**Fig. 3:**
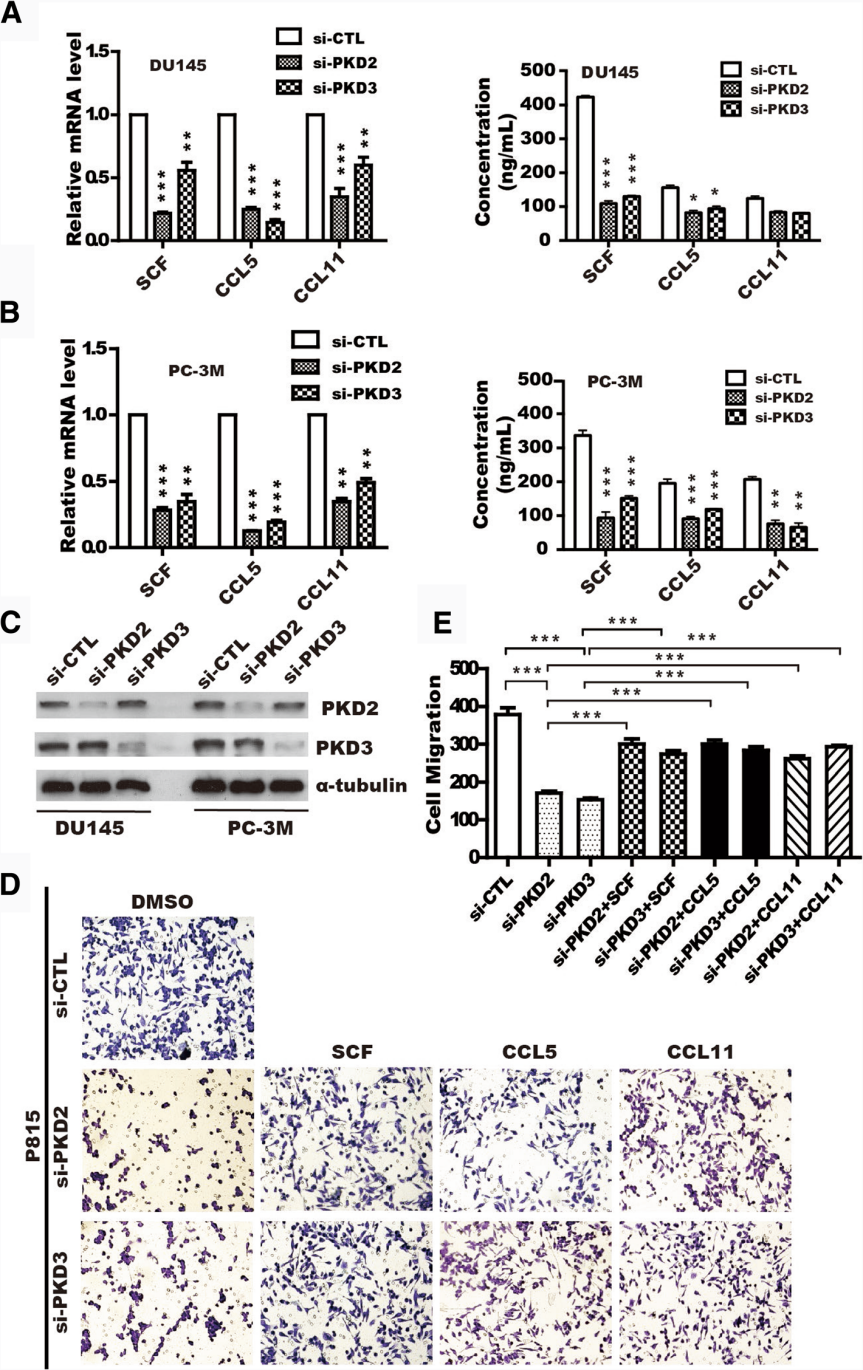
PKD2/3 enhance MCs migration through upregulation of SCF, CCL5 and CCL11 in prostate cancer cellsmRNA and the protein level of the *scf*, *ccl5* and *ccl11* were analyzed by real-time qPCR and ELISA in DU145 (**a**) and PC-3 M (**b**) cells transfected with siRNA of PKD2, PKD3. Data representing the means ± S.D. of three independent experiments was analyzed by one-way ANOVA for significance versus si-CTL. ***p < 0.001, **p < 0.01, *p < 0.05 versus si-CTL. **c** Knockdown efficiency of PKD2 and PKD3 in prostate cancer cells was verified by Western blotting. **d** DU145 cells were transfected with siRNA of PKD2, PKD3, the Conditional medium (CM) was collected to measure migration of P815 cells in response to SCF, CCL5, and CCL11 treatment by transwell assay. **e** Quantification were analyzed from data in D. ***p < 0.001 versus si-CTL by *One-ANOVA* tests

Given that multiple autocrine or paracrine chemokines such as SCF, CCL5 and CCL11 mediated MCs migration and recruitment [23, 36, 37], so we verified whether PKD2 or PKD3 could regulate these chemokines expression in the prostate cancer cells. RT-qPCR showed that mRNA level of *scf*, *ccl5*, and *ccl11* was significantly suppressed in DU145 (Fig. 3a, left panel) or PC-3 M cells (Fig. 3b, left panel) with knockdown of PKD2 or PKD3. Similarly, ELISA assay also found that PKD2 or PKD3 depletion markedly decreased the secretion of SCF, CCL5 and CCL11 in DU145 (Fig. 3a, right panel) or PC-3 M cells (Fig. 3b, right panel). Western blotting was performed to confirm knockdown efficiency (Fig. 3c).

To further clarify whether PKD2/3 mediated chemotactic migration of MCs through upregulation of SCF, CCL5, and CCL11 in prostate cancer, we performed migration assay for P815 MCs in a transwell plate. As expected, chemotactic migration of P815 MCs was inhibited by the CM from DU145 cells with PKD2 and/or PKD3 silencing, while these effects were rescued by the addition of SCF, CCL5 and CCL11 in the CM, respectively (Fig. 3d), specifically, depletion of PKD2 or PKD3 led a significant reduce to only 40% of control group(Fig. 3e). In line with this, similar effect was also observed in PC-3 M cells (Additional file 8: Figure S6A-B). Moreover, SCF, a major chemotactic factor of MCs, drastically reversed inhibition of BMMCs caused by PKD2/3-depletion in DU145 cells (Additional file 8: Figure S6C-D), and knockdown effect of PKDs were verified by immunoblotting (Additional file 8: Figure S6E). Taken together, these data suggested that PKD2/3-regulated SCF, CCL5 and CCL11 secretion in prostate cancer cells may be the key factors that mediated migration and recruitment of MCs in tumor microenvironment.

### PKD2/3 interacts with Erk1/2 and activates Erk1/2 or NF-κB signaling pathway in prostate cancer cells

**Fig. 4:**
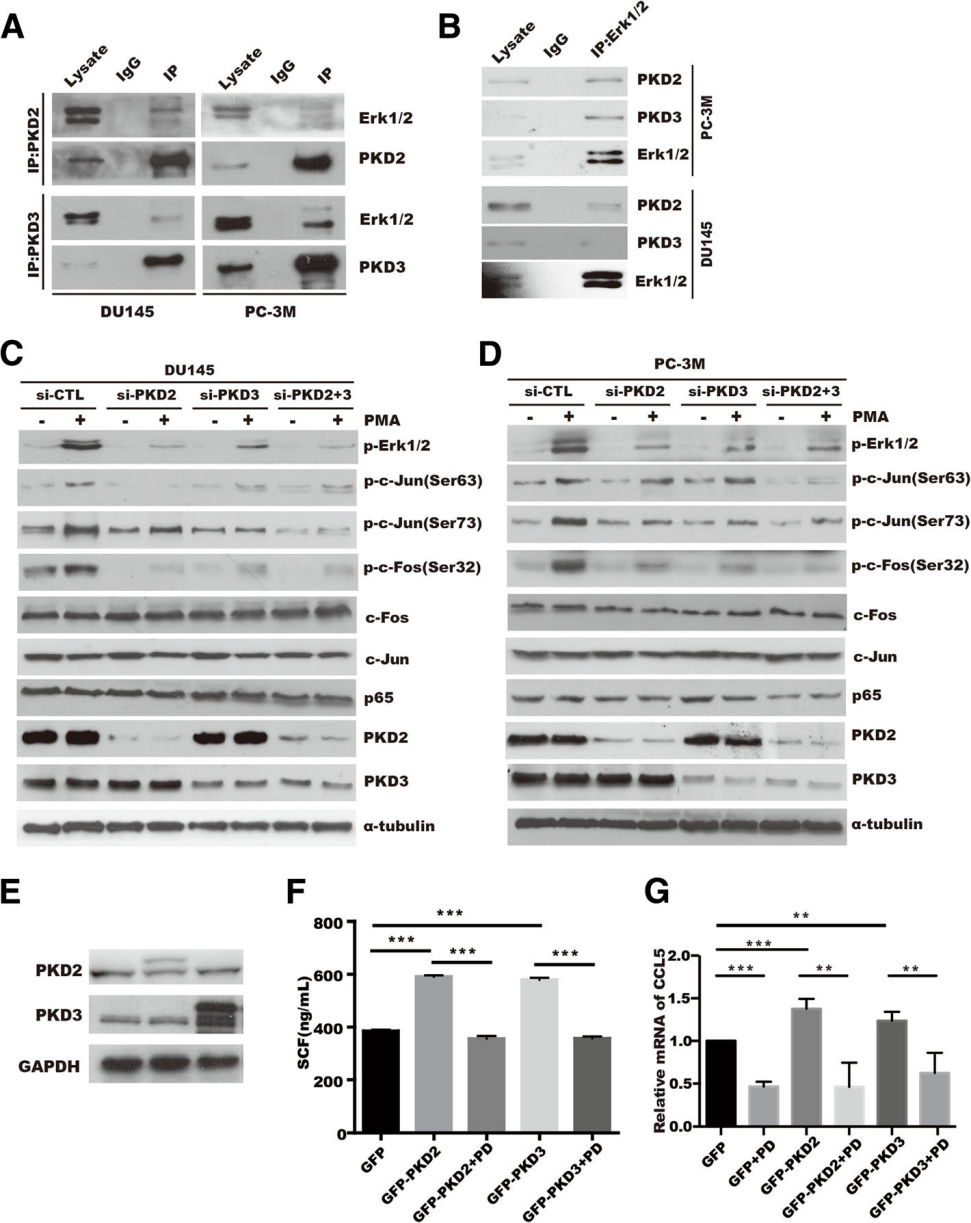
PKD2 and PKD3 promote SCF, CCL5 and CCL11 expression through Erk1/2 signaling pathways. **a-b** Interaction of PKD2 or PKD3 with Erk1/2 was performed by co-IP assay in PC-3 M or DU145 prostate cancer cells. **c-d** DU145 cells (**c**) or PC-3 M cells (**d**) were transfected as indicated and treated with 100 nM PMA, phosphorylation and protein expression were detected by western blotting. **e** Overexpression efficiency of PKD2 and PKD3 in prostate cancer cells was verified by Western blotting. **f** ELISA were applied to measure SCF in conditional medium from DU145 cell transfected with GFP, GFP-PKD2, and GFP-PKD3 in present with or without Erk inhibitor PD98059(PD) treatment. **g** Real-time PCR was performed to analyze *ccl5* expression in DU145 transfected with *GFP, GFP-PKD2* and *GFP-PKD3* plasmids followed by treatment with or without Erk inhibitor PD98059

We previously demonstrated that PKD2 and PKD3 interact with IKKβ, and mediate the pIκB kinase (pIKK)-pIκB-IκB degradation cascade in prostate cancer cells [35]. Moreover, NF-κB and Erk1/2 played a critical role in the regulation of cytokines and chemokines production [38, 39]. To examine whether Erk1/2 signaling is required for PKD2/3 induced -SCF, CCL5, and CCL11 expression in tumor cells, we first analyzed the interaction of endogenous PKD2 or PKD3 with Erk1/2 in prostate cancer cells. Co-immunoprecipitation assay showed that endogenous PKD2 or PKD3 interacted with Erk1/2 in DU145 and PC-3 M prostate cancer cells (Fig. 4a-b), but not with p38 (Additional file 9: Figure S7).

We then explored the effect of PKD2 or PKD3 on the activation of Erk1/2 or NF-κB signaling in response to PKDs agonist PMA in prostate cancer cells. As shown in Fig. 4c-d, basic phosphorylation of Erk1/2 and its downstream target AP-1(c-Jun:c-Fos) axis were slight reduced, while silencing of PKD2, PKD3 significant decreased activation of Erk1/2-AP axis induced by PMA treatment in DU145 and PC-3 M cells, which is also verified by quantification in DU145 cells (Additional file 10: Figure S8A-D and Figure S8I) or PC-3 M cells (Additional file 10: Figure S8E-H and Figure S8J). Similarly, NF-κB signaling was also inhibited by PKD2/3 silencing in response to PMA (Additional file 10: Figure S8K-L), which was in lined with our previous research [35]. These data suggest that Erk1/2 and NF-κB pathway might be modulated synergistically by PKD2 and PKD3 in prostate cancer cells. Next, we evaluated whether inhibition of Erk1/2 or NF-κB signaling by specific inhibitor antagonize PKD2 or PKD3 -induced chemotactic migration factor expression, such as SCF and CCL5. In prostate cancer cells with overexpressed with PKD2 and PKD3 (Fig. 4e), ELISA and RT-qPCR assay demonstrated that Erk specific inhibitor PD98059 (PD) antagonized the upregulation of SCF (Fig. 4f) and CCL5 expression (Fig. 4g) induced by PKD2 or PKD3 in DU145 cells. Similar result of *scf*, *ccl5* and *ccl11* was obtained in mRNA level by treatment of NF-κB inhibitor BAY11–7082(BAY) (Additional file 11: Figure S9B) and JNK inhibitor SP600125(SP) in PC-3 cells (Additional file 11: Figure S9C-D) and DU145 cells (Additional file 11: Figure S9F-H). Collectively, these data indicate that upregulation of SCF, CCL5, and CCL11 expression triggered by PKD2/3 relies on the Erk1/2 or NF-κB signaling pathway.

### PKD2/3 promotes AP-1 or NF-κB binding to the promoter of SCF, CCL5 and CCL11

**Fig. 5:**
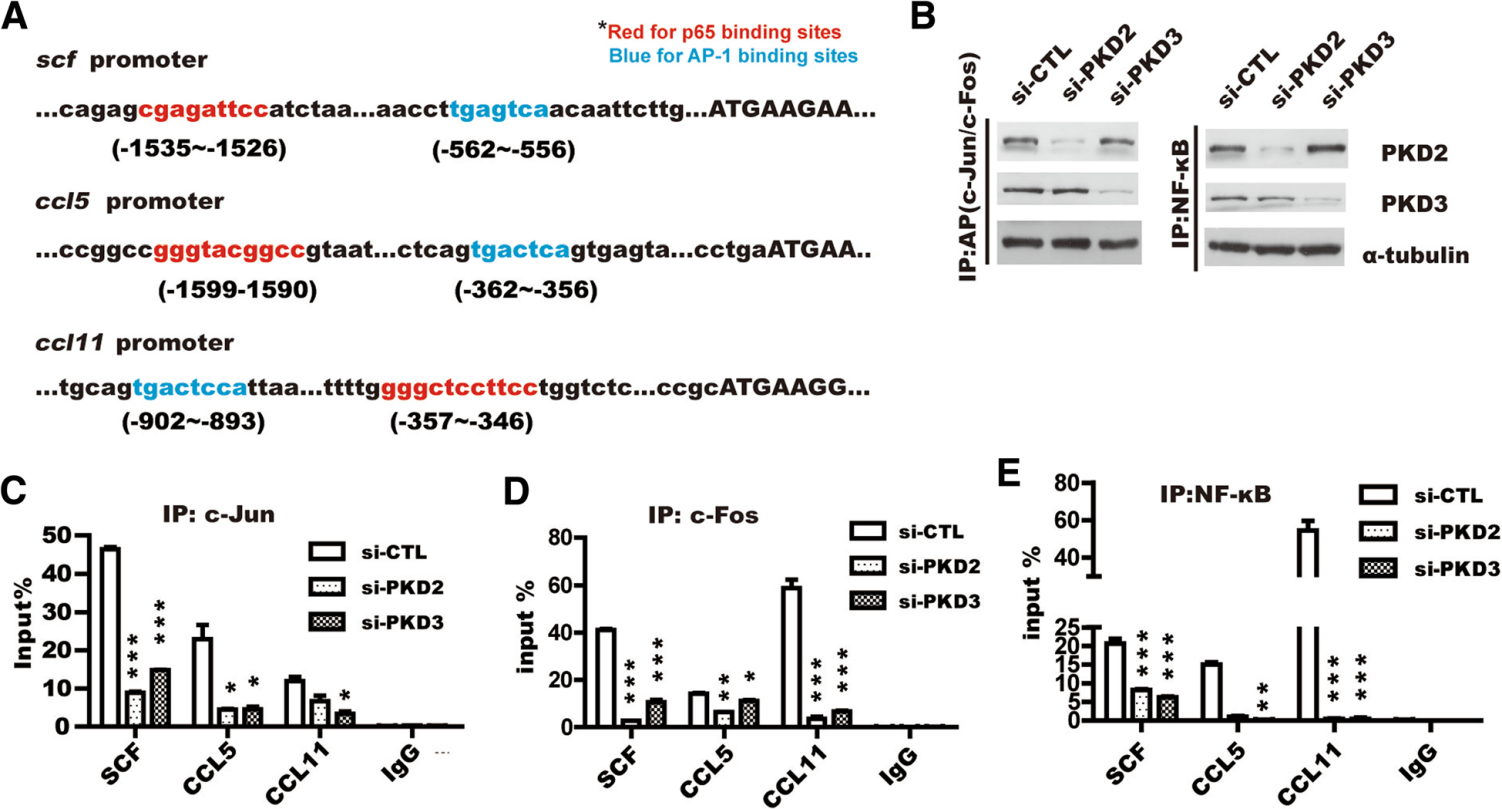
PKD2 and PKD3 are required for SCF, CCL5, and CCL11 transcription. **a** Analysis of the binding site of p65(highlight in red) and AP1(highlight in blue) in the promoters of *scf, ccl5* and *ccl11* using UCSC software online. **b** Western blotting was used to ensure the knockdown effect. ChIP analysis of the binding of c-Jun (**c**), c-Fos (d) and NF-κB (**e**) to the *scf, ccl5* and *ccl11* gene promoter in PC-3 M cells depleted with siRNA of PKD2, PKD3. *Student’s t-test*, *p < 0.05, **p < 0.01 and ***p < 0.001 (*n* = 3). Error bars indicate mean ± S.D.

Given that PKD2/3-mediated Erk1/2 or NF-κB signaling contributes to the upregulation of SCF, CCL5 and CCL11, and AP-1, including c-Jun and c-Fos, is a key transcriptional factor of Erk1/2 signaling [35, 40]. To further investigate whether activation of c-Jun and c-Fos or NF-κB induced by PKD2/3 promotes the binding of c-Jun and c-Fos or NF-κB to the promoter of *scf*, *ccl5* and *ccl11*

genes, we used UCSC online software to identify a putative binding sites of c-Jun and c-Fos or NF-κB in the promoter of *scf*, *ccl5* and *ccl11* genes. Conserved binding sites of p65 and AP-1 existed in the promoter of *scf*, *ccl5* and *ccl11* genes as highlighted with red(p65) and blue(AP-1), respectively (Fig. 5a). Chromatin Immunoprecipitation (ChIP) followed by RT-qPCR showed that PKD2 or PKD3 silencing significantly reduced c-Jun:c-Fos, two subunit of AP-1, binding to the promoter of *scf*, *ccl5*, and *ccl11*, respectively (Fig. 5c and d). Similar effect of PKD2/3 depletion on NF-κB binding to the promoter of above genes was also observed (Fig. 5e). Meanwhile, Knockdown of PKD2 or PKD3 in DU145 cells was verified by Western blotting (Fig. 5b). Taken together, these results indicate that AP-1 and NF-κB play a central role in the transactivation of *scf*, *ccl5* and *ccl11* induced by PKD2 or PKD3 in prostate cancer cells.

### Inhibition of PKD represses MCs infiltration, tumor angiogenesis and tumor growth in vivo

**Fig. 6:**
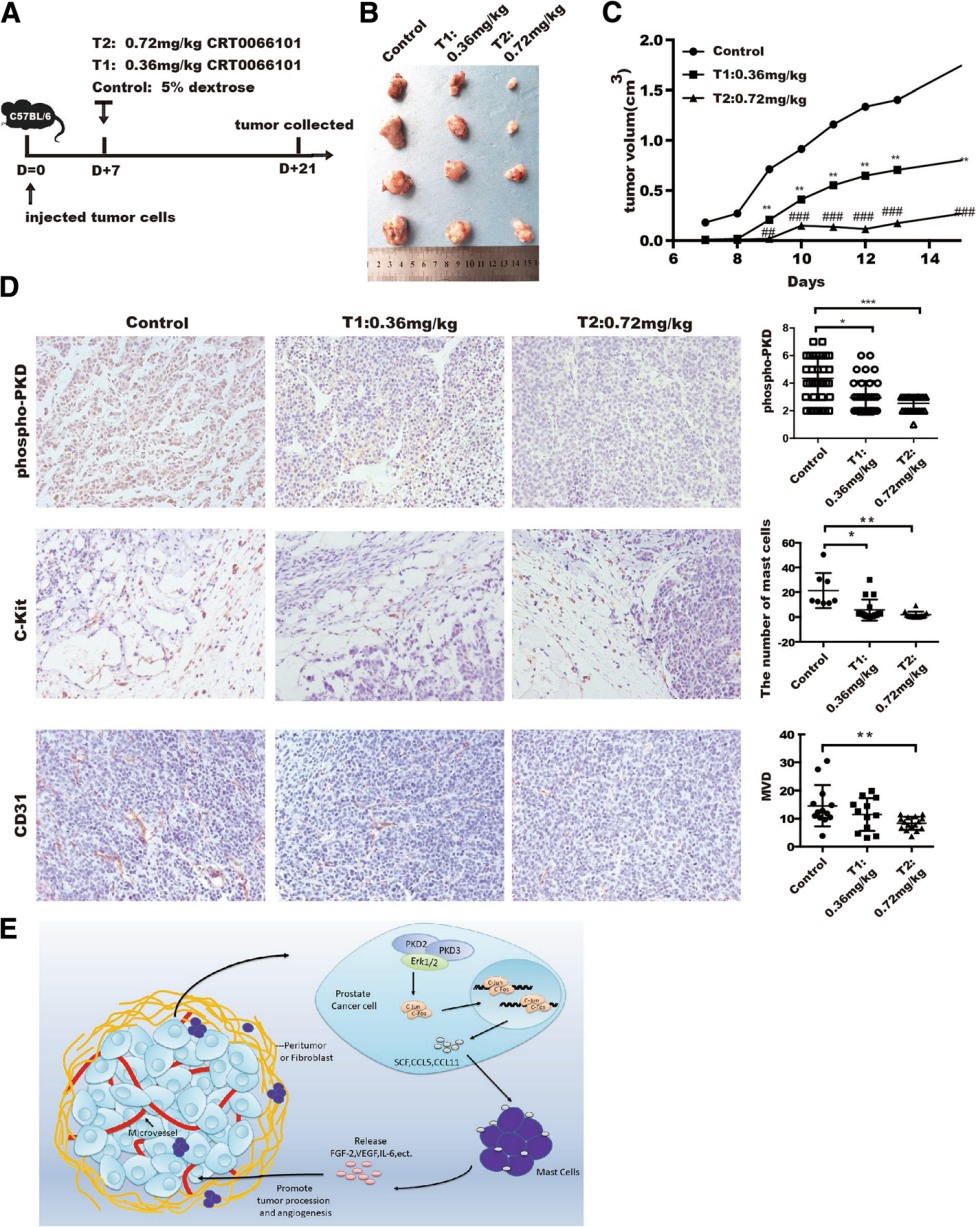
CRT0066101 reduces MCs recruitment and tumor angiogenesis in vivo. **a** Experimental setting. **b** C57BL6 mice bearing RM1 tumors were administered a daily vehicle [control group; 5% (*w*/*v*) dextrose] or CRT0066101 at 20 μM and 40 μM for 2 weeks (4 mice per group), then excised tumor images. **c **Tumor volume were represented at indicated day. **d** Immunohistochemistry staining for phospho-PKD, microvessel density (stained with CD31), and mast cells (stained with c-Kit). Representative image were shown in 400X under the microscope (Left panel). Quantification of the indicated parameter was analyzed among groups after treatment with CRT0066101 (Right panel). **e** Schematic model of the mechanistic role of PKD2 and PKD3 in tumor angiogenesis by regulating SCF-, CCL5-, and CCL11-mediated mast cell recruitment

CRT0066101, a newly developed PKD inhibitor, has been demonstrated to inhibit cancer growth in multiple cancer models [41, 42]. To provide insights into the effect of PKD2/PKD3 on the recruitment of MCs, tumor angiogenesis and tumor growth in prostate cancer model*,* mouse prostate cancer RM1 cells were injected subcutaneously into C57BL/6 mice, mice with RM1 tumors were randomized to three groups as described in method (Fig. 6a). After 2 weeks of treatment, both tumor size (Fig. 6b) and tumor volume (Fig. 6c) were inhibited by CRT0066101 inhibitor, whereas body weight was no changed even at the highest dose of CRT0066101 (Additional file 12: Figure S10). Moreover, CRT066101 treatment resulted in a dose-dependent suppression of PKD activity (phospho-PKD), MC recruitment (c-Kit), and the level of MVD (Fig. 6d and Additional file 1: Table S6). Taken together, these data provide evidence that PKD2 and PKD3 could promote prostate cancer progression through the recruitment of MCs and tumor angiogenesis in prostate cancer microenvironment.

## Discussion

In this study, we have demonstrated that knockdown of PKD2/3 or inhibition of PKD activity in prostate cancer cells in vitro significantly repressed MCs recruitment through regulating AP-1 or NF-κB mediated transcription of SCF, CCL5 and CCL11, thereby inhibiting angiogenic factors expression in MCs. Enhanced activation of PKD was correlated with tumor angiogenesis in prostate tumor tissues compared with the normal tissues. Moreover, PKD inhibitor CRT0066101 attenuated tumor angiogenesis as well as tumor growth in vivo. These findings indicate PKDs are crucial mediator of MC recruitment in prostate cancer, and could be targeted to inhibit MC infiltration and MC-induced tumor angiogenesis.

Our previous studies have demonstrated a significant role of PKDs in prostate cancer cell invasion [35], proliferation, tumor growth [21] and survival [20]. However, the functional role of PKD in tumor angiogenesis is not fully elucidated. Angiogenesis, which plays an important role in immune response, inflammation and tumor progression, is a critical component of tumor microenvironment and increases oxygen, tumor invasion and migration [43–45]. We revealed a positive correlation of PKD activation with MCs recruitment and angiogenesis in prostate cancer.

Interestingly, our pervious results showed that both PKD2 and PKD3 have failed to alter VEGF mRNA expression [35]. In the present study, we found that PKD2 and PKD3 did not directly promote the tube formation of endothelial cells using co-culture with HUVEC cells directly, which imply that PKD2 and PKD3 may promote angiogenesis by remodeling tumor microenvironment. MCs are present in 95.9% of the tumor samples [46, 47], showing versatile roles from dangerous promoters, innocent bystanders, to essential guardians of tumors, depending on the stage and origin of carcinoma [9]. High intratumoral mast cell density is associated with favorable tumor characteristics and good prognosis in prostate cancer [15, 48], oppositely high peritumoral mast cells density process tumor cell proliferation, neo-angiogenesis, invasion and metastasis [47, 49]. Also, our finding showed that PKD2/3 of prostate cancer cells was involved in MCs recruitment and angiogenic factors expression in MCs leading to angiogenesis. Furthermore, Inhibition of PKD by CRT0066101 significantly reduced microvessel density in vivo*,* which are largely attributed to reduce MCs migration. Nevertheless, a recent report showed that pure presence of mast cells in tumor microenvironment does not necessarily have any functional impact on tumor growth, tumor progression or tumor vascularization [50]. These controversial findings remind us that it is of importance to seek specific marker for clarification of intratumoral and peritumoral mast cells as well as corresponding function in tumor microenvironment.

Much more evidences supported that PKD2/3 promoted inflammation and tumor procession in carcinoma and its microenvironments by regulating different cytokine expression and secretion. PKD2 regulated tumor migration and invasion via MAPK pathway, p53-dependent and -independent pathways in tumor progression [22, 51]. Meanwhile, PKD3 was an inconspicuous isoform in PKDs family, which not only regulates tumor invasion in breast cancer but also contributes to HIV-1 provirus binding to the promoter of NF-κB [52]. The current study indicated that PKD2 and PKD3 have a critical role in the regulation of SCF, CCL5, and CCL11 expression, which are pivotal for MC infiltration [23, 36]. Given that PKD2 and PKD3 contributions to NF-κB signaling has been well documented in our previous work. Depletion of PKD2 and PKD3 in prostate cancer cells significantly decreased binding of p65, c-Jun and c-Fos to the *scf*, *ccl5*, and *ccl11* promoter. Collectively, these results indicated that PKD2/3-NF-κB and PKD2/3-Erk1/2 axes played an important role in prostate cancer cell.

## Conclusions

In summary, the current study showed that PKDs serve as a pro-angiogenic molecular by recruitment of mast cells in tumor microenvironment of prostate cancer. We demonstrated that PKD2 and PKD3 played a critical role in the regulation of tumor angiogenesis and defined a novel model in which PKD2 and PKD3 activate Erk1/2 and NF-κB in prostate cancer cells through promoting SCF, CCL5, and CCL11 expression, which further promote MCs recruitment and expression of angiogenic factors consequently, leading to tumor angiogenesis (Fig. 6e). Most importantly, inhibition of PKD2 and PKD3 largely blocked tumor growth and angiogenesis in vivo. This finding not only provided further support for the notion that cancer cells shape a tumor microenvironment that is favorable for facilitating tumor progression, but also added a novel molecular link between tumor biology and tumor angiogenesis.

## Additional files

Additional file 1: **Table S1-S6.** (DOCX 18 kb)

Additional file 2: Supplementary data. (DOCX 21 kb)

Additional file 3: **Figure S1.** mRNA level of PKD1/2/3 expression from prostate cancer TCGA data. (PDF 64 kb)

Additional file 4: **Figure S2.** Autophosphorylation of PKD at s916 in prostate tissue. (PDF 1121 kb)

Additional file 5: **Figure S3.** PKD2/3 in prostate cancer cells promoted chemotactic migration of mast cells. (PDF 1025 kb)

Additional file 6: **Figure S4.** Effect of PKD2 and PKD3 derived prostate cancer cells on endothelial cells tube formation in vitro. (PDF 832 kb)

Additional file 7: **Figure S5.** PKD2/3 silencing of prostate cancer cells reduced angiogenic factor expression in P815 MCs cells. (PDF 785 kb)

Additional file 8: **Figure S6.** SCF, CCL5, and CCL11 rescued MCs migration inhibited by CM from PC-3M cells with PKD silencing (PDF 2500 kb)

Additional file 9: **Figure S7.** PKD2/3 did not interact with p38. (PDF 466 kb)

Additional file 10: **Figure S8.** PKD2/3 modulated Erk1/2 and NF-κB activity in prostate cancer cells in response to PMA. (PDF 812 kb)

Additional file 11: **Figure S9.** NF-κB and JNK inhibitor antagonized SCF, CCL5 and CCL11 mRNA level induced by PKD2 or PKD3 overexpression in DU145 cells (PDF 1352 kb)

Additional file 12: **Figure S10.** Effect of PKD inhibitor on body weight change in vivo. (PDF 514 kb)

## Abbreviations

BMMCs: Bone marrow derived mast cells
CCL11: Chemokine ligand 11
CCL5: Chemokine ligand 5
Erk: Extracellular regulated protein kinases
FGF-2: Fibroblast growth factor
IKKα/β: Inhibitor of nuclear factor kappa-B kinase
IL: Interleukin
IκB: Inhibitor of nuclear factor kappa-B
MCs: Mast cells
NF-κB: Nuclear factor kappa-B
PKC: Protein kinase C
PKD: Protein kinase D
PMA: Phorbol-12-myristate-13-acetate
SCF: Stem cell factor
TNF-α: Tumor Necrosis Factor
VEGF: Vascular endothelial growth factor
