# Supplementary material for: Protein kinase Ds promote tumor angiogenesis through mast cell recruitment and expression of angiogenic factors in prostate cancer microenvironment
Source: J Exp Clin Cancer Res. 2019 Mar 6;38:114. doi: 10.1186/s13046-019-1118-y (PMC6404326; doi:10.1186/s13046-019-1118-y)
Supplement: Supplementary file 1 — Table S1-S6. (DOCX 18 kb) [file 13046_2019_1118_MOESM1_ESM.docx]

**Table S1: siRNA sequence targeted PKDs and VEGF**

| PKDs | Gene ID | 5’-3’ |
| --- | --- | --- |
| si-PKD2 | 25865 | CCUGAGUGUGGCUUCUACGGCCUUU |
| si-PKD3 | 23683 | GAACGAGUCUUUGUAGUAATT |
| si-PKD2 | 101540 | CCGUUUGCCAGGCUUGCAATT |
| si-PKD3 | 75292 | CCGUGUAUCUUCACCGCAATT |
| si-VEGF-1 | 22339 | CAACAAAUGUGAAUGCAGA |
| si-VEGF-2 | 22339 | CUGAUGAGAUCGAGUACAU |

**Table S2: Primer sequence of genes for real-time qPCR**

| Genes | Gene ID | Forward (5’-3’) | Reverse (5’-3’) |
| --- | --- | --- | --- |
| *SCF* | 4254 | GTGGATGACCTTGTGGAGTG | TTGAAGGCATCAATGGATCT |
| *CCL5* | 6352 | AAGGAAGTCAGCATGCCTCT | TTTGCCAGTAAGCTCCTGTG |
| *CCL11* | 6356 | ATCTCCAACTCCAAAGCCAT | TGCCCTTTGGACTGATAATG |
| *TNF-α* | 21926 | ATGAGAAGTTCCCAAATGGC | CTCCACTTGGTGGTTTGCTA |
| *IL-6* | 16193 | AGTCCGGAGAGGAGACTTCA | ATTTCCACGATTTCCCAGAG |
| *IL-8* | 20309 | TTGCCAAGGAGTGCTAAAGAA | GCCCTCTTCAAAAACTTCTCC |
| *IL-10* | 16153 | CCCAGAAATCAAGGAGCATT | TCACTCTTCACCTGCTCCAC |
| *FGF-2* | 14173 | CAACCGGTACCTTGCTATGA | GTGCCACATACCAACTGGAG |
| *VEGF* | 22339 | CACAGCAGATGTGAATGCAG | CTGCGGATCTTGGACAAAC |
| *UBC* | 7316 | ATTTGGGTCGCGGTTCTTG | TGCCTTGACATTCTCGATGGT |
| *GAPDH* | 14433 | AGGTCGGTGTGAACGGATTTG | TGTAGACCATGTAGTTGAGGTCA |

**Table S3: Gene primer sequences of ChIP assay**

| **Transcription Factor** | **Genes** | **Forward (5’-3’)** | **Reverse (3’-5’)** |
| --- | --- | --- | --- |
| **NF-KB** | *SCF* | GCGCGAGGTATTTCGTCTGT | GCACGGTAAATGCCCCAGAA |
|  | *CCL5* | ATACCGGCCAATGCTTGGTT | TTTATAGGGCCAGTTGAGGGG |
|  | *CCL11* | TGGGTGGGAGCCTAATGGAA | TCCTGTGTTCACATGGTGGTC |
| **AP-1** | *SCF* | CTGTGTTGGCGACCTGTAGT | GGCCATTGTCTATCACCTCT |
|  | *CCL5* | CATGGATGAGGGAAAGGAGGTA | GTGGCAGTTAGGACAGGATCA |
|  | *CCL11* | TTGCAGTACCTCCACACCAG | CAGCCTCTCGTGCCCTTATT |

**Table S4 The number of human tissue microarray.**

| Company | Catalog number |
| --- | --- |
| Alenabio (Xi’an, China) | PR242a；PR242b；[PR243c](http://www.alenabio.com/public/details?productId=29877&searchText=) |

**Table S5: The correlation of tumor stage, MVD, mast cells and phospho-PKD in tissue microarray.**

|  |  | Stage | MVD | phospho-PKD |
| --- | --- | --- | --- | --- |
| Stage | Correlation Coefficient | 1.000 | 0.542^**^ | 0.776^**^ |
|  | Sig. (2-tailed) | - | 0.006 | 0.000 |
| MVD | Correlation Coefficient | 0.542^**^ | 1.000 | 0.509^*^ |
|  | Sig. (2-tailed) | 0.006 | - | 0.011 |
| phospho-PKD | Correlation Coefficient | 0.776^**^ | 0.509^*^ | 1.000 |
|  | Sig. (2-tailed) | 0.000 | 0.011 | - |

*, Correlation is significant at the 0.05 level (2-tailed).**, Correlation is significant at the 0.01 level (2-tailed).

**Table S6: the correlation of phospho-PKD with mast cells and microvessels in tumor tissue of animal model.**

|  |  | phospho-PKD | Microvessels |
| --- | --- | --- | --- |
| phospho-PKD | Correlation Coefficient | 1.000 | 0.294^*^ |
|  | Sig. (2-tailed) | - | 0.043 |
|  | N | 45 | 37 |
| Microvessels  （MVD） | Correlation Coefficient | 0.294^*^ | 1.000 |
|  | Sig. (2-tailed) | 0.043 | - |
|  | N | 37 | 45 |

*, Correlation is significant at the 0.05 level (2-tailed).

**, Correlation is significant at the 0.01 level (2-tailed)
