# Supplementary material for: Protein kinase Ds promote tumor angiogenesis through mast cell recruitment and expression of angiogenic factors in prostate cancer microenvironment
Source: J Exp Clin Cancer Res. 2019 Mar 6;38:114. doi: 10.1186/s13046-019-1118-y (PMC6404326; doi:10.1186/s13046-019-1118-y)
Supplement: Supplementary file 2 — Supplementary data. (DOCX 21 kb) [file 13046_2019_1118_MOESM2_ESM.docx]

**Supplementary data of clinical array:**

| **Legend : PR242a/PR242b** | | | | | | | | | | | |
| --- | --- | --- | --- | --- | --- | --- | --- | --- | --- | --- | --- |
| **Pos.** | **No.** | **Sex** | **Age** | **Organ** | **Pathology** | **Grade** | **Stage** | **Gleason grade** | **Gleason score** | **TNM** | **type** |
| A1 | 1 | M | 66 | Prostate | Adenocarcinoma | 1 | IV | 2 | 1+2 | T3N1M1 | Malignant |
| A2 | 2 | M | 66 | Prostate | Adenocarcinoma | 1 | IV | 2 | 1+2 | T3N1M1 | Malignant |
| A3 | 3 | M | 65 | Prostate | Adenocarcinoma | 2 | II | 3 | 3+3 | T2N0M0 | Malignant |
| A4 | 4 | M | 65 | Prostate | Adenocarcinoma | 2 | II | 3 | 3+3 | T2N0M0 | Malignant |
| A5 | 5 | M | 73 | Prostate | Adenocarcinoma | 1 | II | 2 | 2+2 | T2N0M0 | Malignant |
| A6 | 6 | M | 73 | Prostate | Adenocarcinoma | 1 | II | 2 | 2+2 | T2N0M0 | Malignant |
| B1 | 7 | M | 75 | Prostate | Adenocarcinoma | 1 | IV | 2 | 2+2 | T4N1M1 | Malignant |
| B2 | 8 | M | 75 | Prostate | Adenocarcinoma | 1 | IV | 2 | 2+2 | T4N1M1 | Malignant |
| B3 | 9 | M | 72 | Prostate | Adenocarcinoma | 2 | II | 3 | 3+3 | T2N0M0 | Malignant |
| B4 | 10 | M | 72 | Prostate | Adenocarcinoma | 1 | II | 2 | 3+2 | T2N0M0 | Malignant |
| B5 | 11 | M | 58 | Prostate | Adenocarcinoma | 2 | II | 3 | 3+3 | T2N0M0 | Malignant |
| B6 | 12 | M | 58 | Prostate | Adenocarcinoma | 2 | II | 3 | 3+3 | T2N0M0 | Malignant |
| C1 | 13 | M | 64 | Prostate | Adenocarcinoma | 2 | IV | 4 | 2+4 | T3N0M1b | Malignant |
| C2 | 14 | M | 64 | Prostate | Adenocarcinoma | 2 | IV | 4 | 3+4 | T3N0M1b | Malignant |
| C3 | 15 | M | 70 | Prostate | Adenocarcinoma | 2--3 | II | 4 | 4+4 | T2N0M0 | Malignant |
| C4 | 16 | M | 70 | Prostate | Adenocarcinoma | 2 | II | 4 | 4+3 | T2N0M0 | Malignant |
| C5 | 17 | M | 75 | Prostate | Adenocarcinoma | 2 | IV | 3 | 3+3 | T3N0M1b | Malignant |
| C6 | 18 | M | 75 | Prostate | Adenocarcinoma | 2 | IV | 3 | 3+3 | T3N0M1b | Malignant |
| D1 | 19 | M | 76 | Prostate | Adenocarcinoma | 3 | IV | 5 | 5+4 | T3N1M1b | Malignant |
| D2 | 20 | M | 76 | Prostate | Adenocarcinoma | 3 | IV | 5 | 5+4 | T3N1M1b | Malignant |
| D3 | 21 | M | 27 | Prostate | Adjacent normal prostate tissue | - | - | - | - | - | NAT |
| D4 | 22 | M | 27 | Prostate | Adjacent normal prostate tissue | - | - | - | - | - | NAT |
| D5 | 23 | M | 25 | Prostate | Adjacent normal prostate tissue | - | - | - | - | - | NAT |
| D6 | 24 | M | 25 | Prostate | Adjacent normal prostate tissue | - | - | - | - | - | NAT |

| **Legend : [PR243c](http://www.alenabio.com/public/details?productId=29877&searchText=)** | | | | | | | | |
| --- | --- | --- | --- | --- | --- | --- | --- | --- |
| **Pos.** | **Age** | **Sex** | **Organ** | **Pathology diagnosis** | **TNM** | **Grade** | **stage** | **Type** |
| A1 | 66 | M | Prostate | Adenocarcinoma | T2N0M0 | 2 | IIA | Malignant |
| A2 | 66 | M | Prostate | Adenocarcinoma | T2N0M0 | 2 | IIA | Malignant |
| A3 | 80 | M | Prostate | Saccular ectasia | - | - | - | NAT |
| A4 | 80 | M | Prostate | Saccular ectasia | - | - | - | NAT |
| A5 | 75 | M | Prostate | Adenocarcinoma | T2N1M1C | 2 | IV | Malignant |
| A6 | 75 | M | Prostate | Adenocarcinoma | T2N1M1C | 2 | IV | Malignant |
| A7 | 73 | M | Prostate | Saccular ectasia | - | - | - | NAT |
| A8 | 73 | M | Prostate | Adjacent normal prostate tissue | - | - | - | NAT |
| B1 | 82 | M | Prostate | Adenocarcinoma | T2N0M0 | 3 | IIA | Malignant |
| B2 | 82 | M | Prostate | Adenocarcinoma | T2N0M0 | 3 | IIA | Malignant |
| B3 | 27 | M | Prostate | Hyperplasia | - | - | - | NAT |
| B4 | 27 | M | Prostate | Hyperplasia | - | - | - | NAT |
| B5 | 76 | M | Prostate | Adenocarcinoma | T3N0M0 | 3 | III | Malignant |
| B6 | 76 | M | Prostate | Adenocarcinoma | T3N0M0 | 3 | III | Malignant |
| B7 | 68 | M | Prostate | Hyperplasia | - | - | - | NAT |
| B8 | 68 | M | Prostate | Hyperplasia | - | - | - | NAT |
| C1 | 66 | M | Prostate | Adenocarcinoma | T3AN0M0 | 3 | III | Malignant |
| C2 | 66 | M | Prostate | Adenocarcinoma | T3AN0M0 | 3 | III | Malignant |
| C3 | 21 | M | Prostate | Saccular ectasia | - | - | - | NAT |
| C4 | 21 | M | Prostate | Saccular ectasia | - | - | - | NAT |
| C5 | 66 | M | Prostate | Adenocarcinoma | T2N0M0 | 3 | II | Malignant |
| C6 | 66 | M | Prostate | Adenocarcinoma | T2N0M0 | 3 | II | Malignant |
| C7 | 35 | M | Prostate | Adjacent normal prostate tissue | - | - | - | NAT |
| C8 | 35 | M | Prostate | Adjacent normal prostate tissue | - | - | - | NAT |
