# Supplementary figures and images for: Protein kinase Ds promote tumor angiogenesis through mast cell recruitment and expression of angiogenic factors in prostate cancer microenvironment
Source: J Exp Clin Cancer Res. 2019 Mar 6;38:114. doi: 10.1186/s13046-019-1118-y (PMC6404326; doi:10.1186/s13046-019-1118-y)

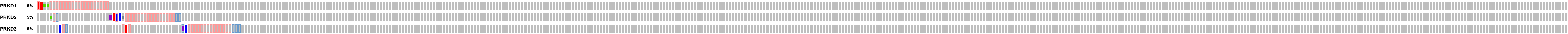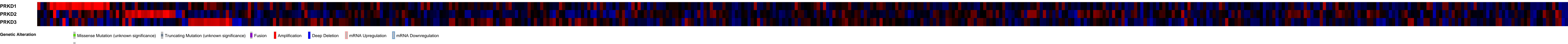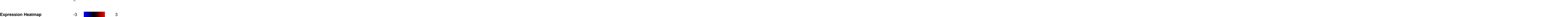

Supplement: Supplementary file 3 — Figure S1. mRNA level of PKD1/2/3 expression from prostate cancer TCGA data. (PDF 64 kb) [file 13046_2019_1118_MOESM3_ESM.pdf]

**Fig. S2**

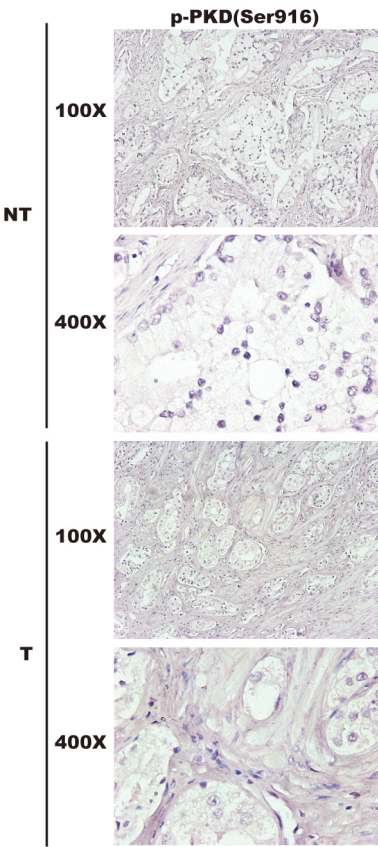

Supplement: Supplementary file 4 — Figure S2. Autophosphorylation of PKD at s916 in prostate tissue. (PDF 1121 kb) [file 13046_2019_1118_MOESM4_ESM.pdf]

**Fig. S3**

**A**

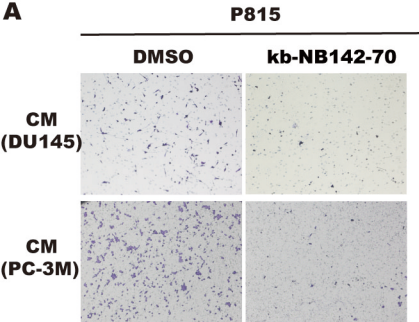

**DU145**

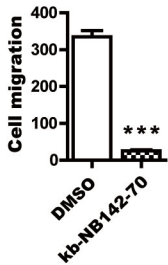

**PC-3M**

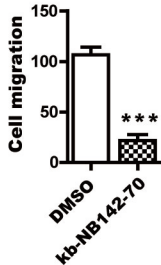

**B**

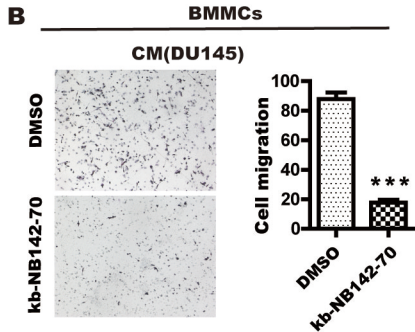

Supplement: Supplementary file 5 — Figure S3. PKD2/3 in prostate cancer cells promoted chemotactic migration of mast cells. (PDF 1025 kb) [file 13046_2019_1118_MOESM5_ESM.pdf]

**Fig. S4****A**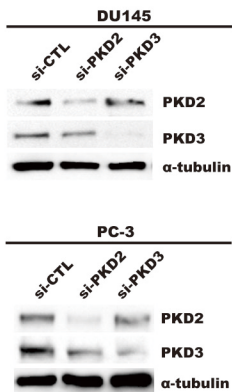**B**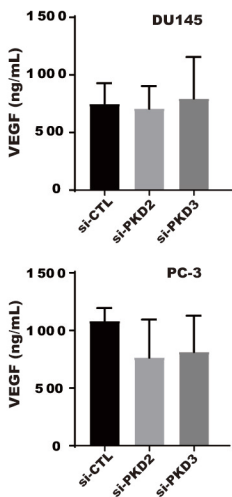**C**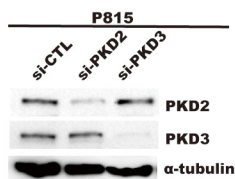**D**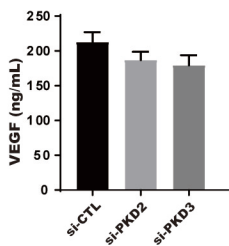**E****DU145(CM)****GFP****PKD2****PKD3**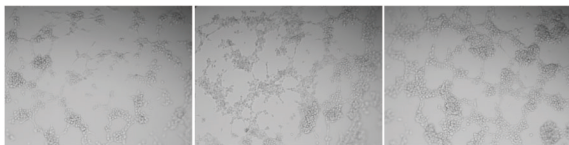**F**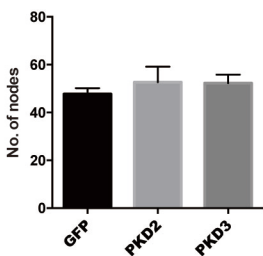

Supplement: Supplementary file 6 — Figure S4. Effect of PKD2 and PKD3 derived prostate cancer cells on endothelial cells tube formation in vitro. (PDF 832 kb) [file 13046_2019_1118_MOESM6_ESM.pdf]

**Fig. S5**

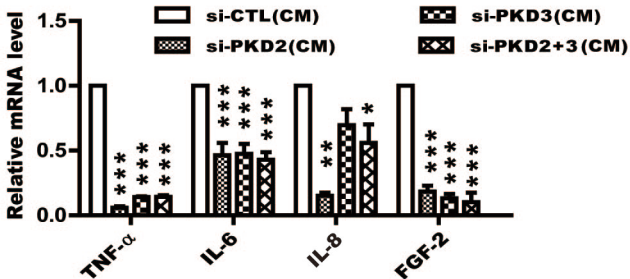

Supplement: Supplementary file 7 — Figure S5. PKD2/3 silencing of prostate cancer cells reduced angiogenic factor expression in P815 MCs cells. (PDF 785 kb) [file 13046_2019_1118_MOESM7_ESM.pdf]

**Fig. S6****A**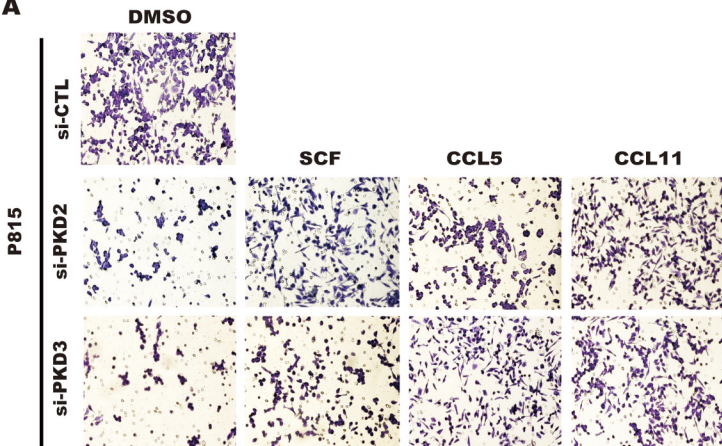**B**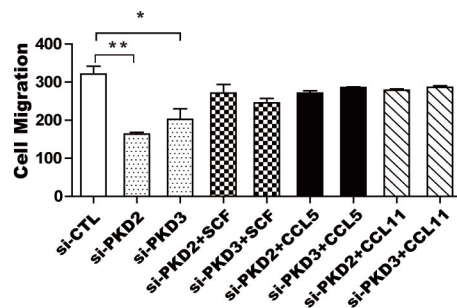**C**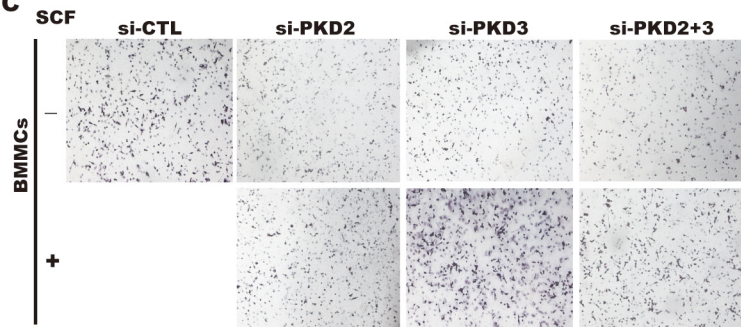**D**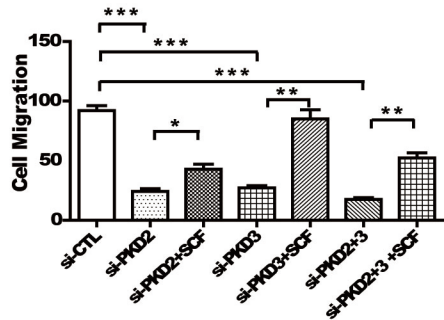**E**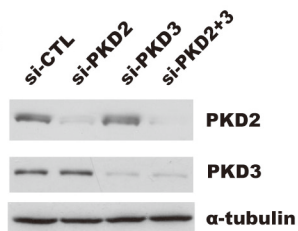

Supplement: Supplementary file 8 — Figure S6. SCF, CCL5, and CCL11 rescued MCs migration inhibited by CM from PC-3M cells with PKD silencing (PDF 2500 kb) [file 13046_2019_1118_MOESM8_ESM.pdf]

**Fig. S7**

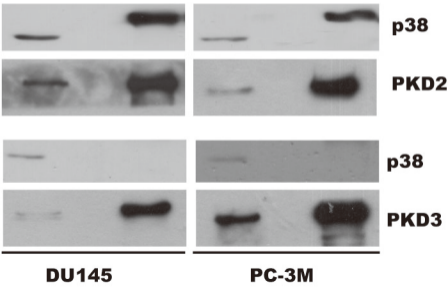

Supplement: Supplementary file 9 — Figure S7. PKD2/3 did not interact with p38. (PDF 466 kb) [file 13046_2019_1118_MOESM9_ESM.pdf]

Fig. S8

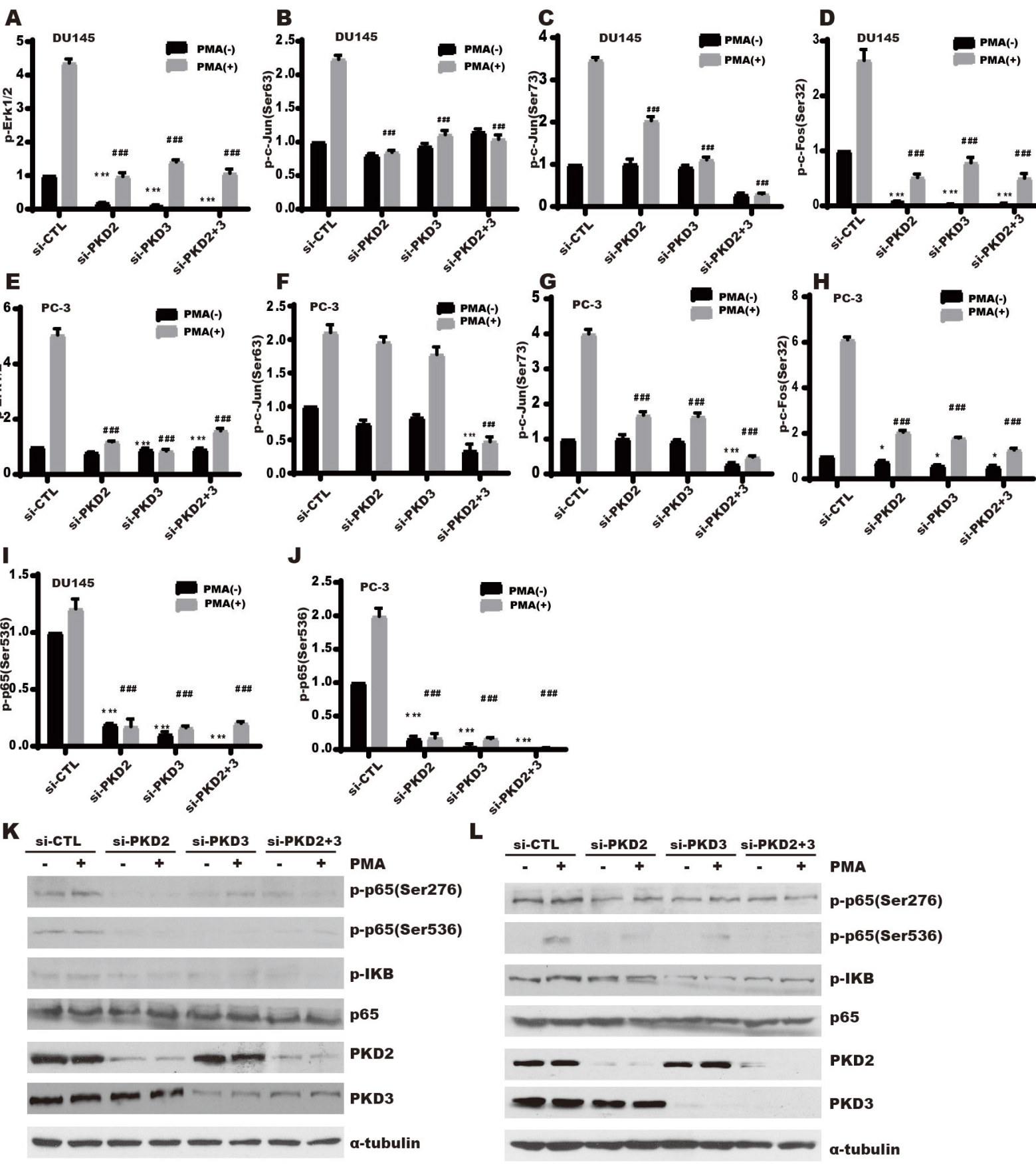

Supplement: Supplementary file 10 — Figure S8. PKD2/3 modulated Erk1/2 and NF-κB activity in prostate cancer cells in response to PMA. (PDF 812 kb) [file 13046_2019_1118_MOESM10_ESM.pdf]

**Fig. S9**

**A**

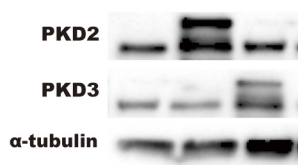

**B**

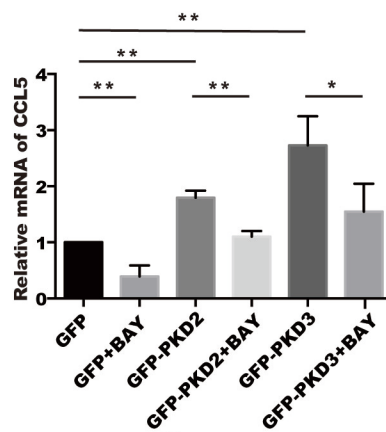

**C**

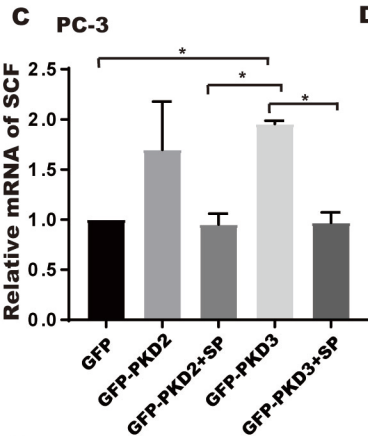

**D**

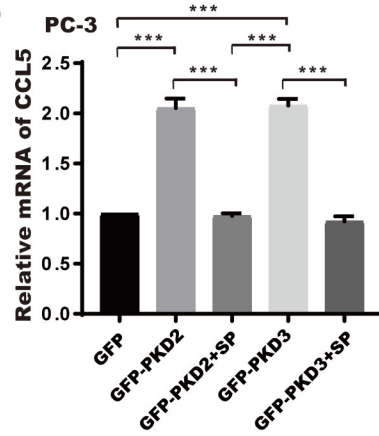

**E**

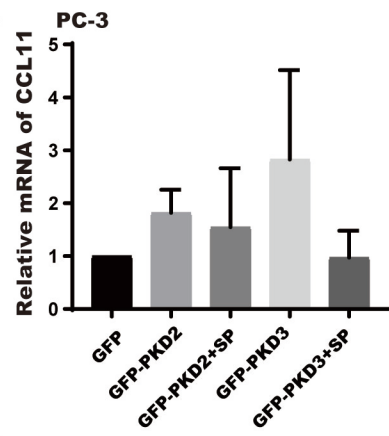

**F**

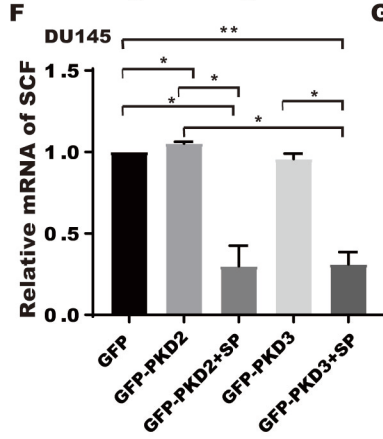

**G**

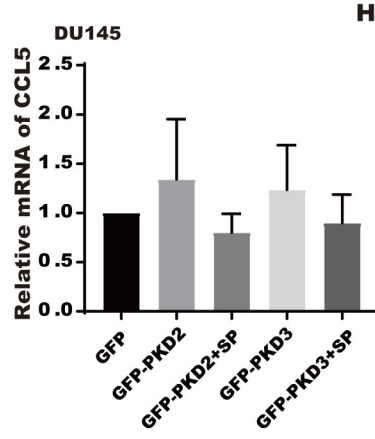

**H**

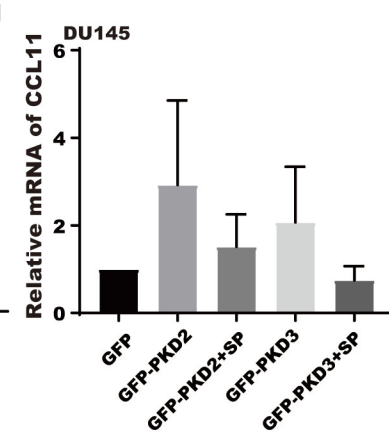

Supplement: Supplementary file 11 — Figure S9. NF-κB and JNK inhibitor antagonized SCF, CCL5 and CCL11 mRNA level induced by PKD2 or PKD3 overexpression in DU145 cells (PDF 1352 kb) [file 13046_2019_1118_MOESM11_ESM.pdf]

**Fig. S10**

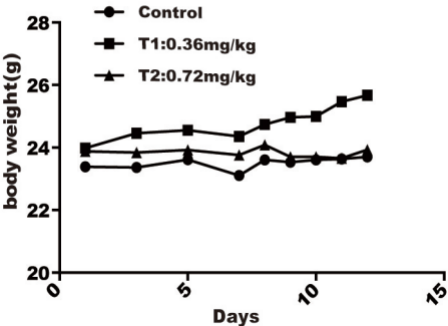

Supplement: Supplementary file 12 — Figure S10. Effect of PKD inhibitor on body weight change in vivo. (PDF 514 kb) [file 13046_2019_1118_MOESM12_ESM.pdf]
